# Dried blood spot 3-year stability suitability for biomarker discovery and biobanking initiatives in companion animals

**DOI:** 10.1371/journal.pone.0352031

**Published:** 2026-08-11

**Authors:** Kelli D. Goodman, Joshua Wilson, Matthew Mitchell, Alison Colyer, Richard Haydock, Brian Keppler, Anne M. Evans, David Allaway

**Affiliations:** 1 Metabolon, Inc., Morrisville, North Carolina, United States of America; 2 Waltham Petcare Science Institute, Melton Mowbray, Leicestershire, United Kingdom; Anhui University of Chinese Medicine, CHINA

## Abstract

Dried blood spot (DBS) sampling is a rapid, cost-effective, and sustainable method that requires minimal processing. It can be performed using remnant blood samples from clinics or via pinprick at home. The DBS metabolome has similar discriminatory potential to the plasma metabolome, making it suitable for biomarker discovery. This study evaluated the suitability of DBS for long-term storage and its potential for biomarker discovery from companion animal (cats and dogs) biobanks using two different approaches. Objective 1 assessed whether two independent metabolic profiling analyses using duplicate DBS card samples, conducted three years apart would provide similar phenotype discrimination. Objective 2 evaluated the potential to combine independent metabolomics datasets through a bridging sample, facilitating insights from prospective biobanks and enabling the reuse of previously acquired data. Using 54 samples from 5 phenotypic classes (1 cat breed and 4 dog breeds), after three years of storage at −80°C, the DBS metabolome showed a 7% reduction (782–728 metabolites). A thorough investigation indicated that this was likely due to experimental variation rather than time-dependent sample instability. Despite data loss, classification remained consistent across independent datasets using various approaches. Hierarchical cluster analysis (HCA) demonstrated correct pairing of individuals between runs for 81% of individuals. Partial least squares-discriminant analysis and Random Forest (RF) utilized many of the same metabolites to discriminate between classes and RF also accurately predicted blinded samples with similar probability in both runs. RF and HCA analyses of merged data performed well, with RF correctly predicting breed for two blinded samples and HCA correctly pairing 76% of individuals. These findings indicate that quality control samples can be used to normalize independent metabolomics studies to a similar baseline enabling combined analysis at a future time. Overall, the data support DBS as a suitable sample for several years of storage in biobanks, with minimal data loss, making it viable for biomarker discovery. Implementing DBS sampling of remnant blood can accelerate research activities, and merging data through suitable bridging samples can enhance the reuse of metabolome data, providing additional biological and economic value.

## Introduction

There is a need for a rapid, inexpensive, and sustainable approach to acquire biological samples suitable for biobanking to accelerate biomarker discovery in veterinary health sciences [1]. Dried blood spot (DBS) sampling is a relatively rapid method, taking less than 3 minutes to collect and store the sample; it is inexpensive (a DBS card costs less than 3 US dollars), does not require an in-clinic visit or phlebotomist, and is a sustainable methodology that requires very low sample volume and does not require laboratory equipment to process blood to plasma/serum. It can also be used to obtain biological information from remnant blood samples in clinics or from in-home pinpricks with minimal processing [2]. In a veterinary setting, companion animals may have blood samples taken from which remnant samples could be processed and stored with minimal resources. With appropriate validation [3], DBS samples can enable rapid expansion of a biobank, providing opportunities to identify candidate biomarkers for many diseases [4], notably including rare diseases. The plasma metabolome is often used for biomarker discovery, given that it provides information related to genetics, health state, and environment [2,5,6]. DBS includes plasma and, due to its cellular components, additional information from structural lipids and intracellular metabolism is obtained. For veterinarian researchers/clinicians to invest actively in a DBS biobank initiative, they require confidence that DBS samples acquired over several years provide reliable, comparable data [7]. These data may be acquired across multiple years when used in retrospective studies or may be acquired prospectively on a more regular basis.

Stability of the DBS metabolome has been examined in non-targeted studies with varied reported success at different temperatures. DBS samples have been reported to be stable when stored at −20°C for up to 6 months, with 4–5% of analytes reported to be unstable between 6–12 months [8]. A comparison of DBS samples from a cohort stored for between 1–10 years at −20°C and analysed in a single batch demonstrated a ~ 29% reduction in analyte abundance associated with storage time [9]. Stability at different temperatures for up to 6 months has been evaluated, and −80°C has been recommended [10]. However, to our knowledge, no study has demonstrated stability of the DBS metabolome from the same sample extracted at separate times, for periods greater than 1-year at −80°C, using untargeted discovery methodology. This proof of principle study was designed to develop appropriate methodologies and test these possibilities.

**Fig 1:**
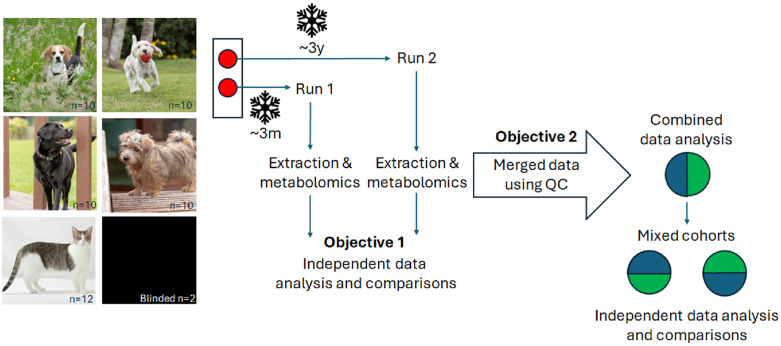
DBS stability study design overview. Metabolite profiling was performed on duplicate DBS samples collected from 54 individuals, using the same platform approximately 3 years apart. Objective 1 compared the results of the independent data analysis from the two runs. Objective 2 used a DBS QC sample that was assayed on both occasions to merge data from both runs prior to analysis. Suitability of the effectiveness of merging data was tested by generating two synthetic data sets, generating cohorts with each run providing half of the dogs. These were then used to assess the ability of merged data to model and accurately predict the class of samples in a separate data set.

We previously reported that companion animal DBS samples were similar to their matched plasma samples in their ability to discriminate between classes (using species (cat/dog), dog breed size and dog breed) [1]. Using additional DBS cards collected at the same time, here we report an untargeted metabolome analysis using duplicate spots on the same profiling platform, analysed 3-years apart. We had two objectives with the analysis: 1) to determine whether the ability to discriminate between classes declined with storage time, and 2) to evaluate the suitability of merging DBS datasets using a DBS Quality Control (QC) sample extracted in both sets (Fig 1).

## Materials and methods

### Ethics statement

The study was approved by the WALTHAM Animal Welfare and Ethical Review Body (AWERB 79726). Fifty-four (54) animals (minimum of 10 per cat/dog breed, also referred to as cat/dog class) housed at the WALTHAM Petcare Science Institute according to the code of practice, which sets out the standards of care and accommodation of animals required by the Animals (Scientific) Procedures Act 1986, took part in the study (Refer to the Animals section below for breed specifics).

### Study design

The minimum number of 10 individuals per class was based on successful discrimination of class using the plasma metabolome using that number [11,12]. During each animal’s biannual health check, an additional blood sample (1mL) was drawn to provide a plasma sample (250 µL) and five DBS cards (each card having 5 x 20 µL spots) to compare plasma and DBS metabolomes for class discrimination as published in [1]. For this study, the DBS cards were maintained under typical veterinary clinic conditions (4°C for 72 hours) prior to long-term storage at −80°C. While plasma was collected, the data was not analysed as part of this study.

### Animals

Clinically healthy adult dogs and cats (n = 54, age range 1–9 years), representing each of four dog breeds of three sizes (n = 10 Labrador retriever – large, n = 10 Beagle – medium, n = 10 Petit Basset Griffon Vendeen (PBGV) – medium, and n = 10 Norfolk terrier – small) and the British Domestic Shorthair cat (DSH, n = 12) on their standard maintenance diet were recruited into the study. All groups were balanced for sex (1:1 male:female). On two occasions, the corresponding plasma samples were not prepared within the required timelines, and two additional animals were sampled to provide both plasma and DBS samples. The DBS samples from the two animals (n = 2) for which plasma was not available were provided as “blinded” samples to analysts and statisticians, allowing an ad hoc test of the ability to assign these two unknown samples to the correct classes (species and if required, breed size and breed).

### Blood sampling and sample preparation

A fasted (>12 hours) blood sample (1mL), from the jugular venipuncture site, was collected into a lithium heparin tube and kept on ice until receipt in the laboratory (time to lab was < 30 minutes). For DBS, blood (0.5 ml) was drawn from the lithium heparin tube using a multi-pipettor and aliquots (20 µL) were applied to individual sites on Whatman™903 Protein Saver cards (5 spots per card, 5 cards) (Product Number: 11962089). Blood spots were air-dried at room temperature for 3 hours prior to being individually sealed in Whatman™903 Foil-Barrier Sample Bags (Product Number: 11984265) with a Whatman™ FTA™ Desiccant Packet (Product Number: 10111442). The cards were stored at 4°C for ~72 hours prior to storage at −80°C. A set of DBS cards (one per animal, 5 spots total) was sent on dry ice to Metabolon, Inc. for metabolomic profiling, where cards were stored at −80°C until the first sample processing analysis (two punches were utilized), then were returned to −80°C storage for an additional 3-years until the second sample processing analysis (two additional punches were utilized). The final spot on the card was not utilized.

In addition to the study samples, a human DBS QC sample was prepared in bulk at Metabolon, Inc. four years earlier by spotting 25 µL of pooled human K2EDTA whole blood (purchased from BioIVT) onto Whatman™903 Protein Saver cards (5 spots per card, 200 cards) (Product Number: 11962089). Blood spots were air-dried at room temperature for 24 hours prior to being sealed in gas-impermeable bags (Product Number: 11984265) (5 cards per bag) containing two 5 g color-indicating desiccant packs (Pillow Pak, Product Number: AS-IND-2GR) and then stored at −20°C for long-term storage.

### Sample processing

DBS samples were acquired between January and November 2020 and were maintained at Metabolon, Inc. for analysis at −80°C in individual foil bags containing one desiccant packet. DBS cards were thawed once for the first analysis on February 12, 2021, then were resealed in foil bags (desiccant packets were replaced as needed) and returned to −80°C. Samples were thawed again approximately 3 years later for the second analysis at Metabolon, Inc. on December 18, 2023, using different spots from the same cards. Samples were maintained at −80°C except during each extraction, where they were stored at room temperature (~1–2 hours). At each analysis (2021 and 2023), four human DBS QC cards (preparation described above) were thawed and analysed concurrently with the study samples according to the method described below.

Samples were processed according to validated methods [13–15] with certain modifications made for DBS. Briefly, two 6 mm punches were taken from each DBS card (one punch was taken from the middle of two spots) and were plated together in a 2 mL deep-well plate and rehydrated with 150 µL of water. After 2 minutes of vigorous shaking on a SPEXC 2000 Geno/Grinder at 350 strokes per minute (spm), protein was precipitated with 500 µL of a methanol solution containing isotopically-labeled internal standards to assess recovery for quality control (QC) purposes. After another vigorous shake (4 minutes at 500 spm) and centrifugation (2800 rpm for 10 minutes at 15°C, the supernatant was divided into five fractions: one for each of the four analytical methods and one reserved for backup if needed. The five plates were placed under a steady flow of nitrogen (SPE-DRY 96) to remove all organic solvent then stored overnight under nitrogen before preparation for analysis.

### Additional quality control measures

In addition to study samples, each extraction plate contained three process blanks (blank DBS punches), four replicates of a human DBS QC sample (preparation described above), and a highly characterized human plasma QC sample (purchased from BioIVT) in singlet to aid in data QC. These eight samples were extracted concurrently with the study samples using the same preparation method described above. DBS blanks were included in every set to ensure biochemicals detected in the biological samples met a 3:1 signal-to-noise ratio over blank levels. The human DBS QC sample was included to monitor analytical variability of detected biochemicals. The inclusion of the same DBS QC sample in both sample sets (analysed years apart) enabled bridging (merging) the data sets. Experimental samples were injected in randomized order within a defined QC sample template, where QC samples were evenly distributed throughout the analysis.

### Ultrahigh performance liquid chromatography-tandem mass spectrometry (UPLC/MS-MS)

Untargeted UPLC-MS/MS analysis of biochemicals was performed on samples extracted from DBS samples by Metabolon, Inc. according to published methods [13–15]. Detailed method descriptions including gradients, solvent conditions, instrument parameters, and standards used can be found in [15] supplementals. Briefly, each of the four dried extract aliquots was reconstituted in a solvent compatible with the chromatographic method for which it was destined, and each solvent contained a set of labelled internal standards to monitor injection sensitivity, signal reproducibility, m/z accuracy, and chromatographic consistency. Two aliquots were analysed under acidic conditions in positive electrospray ionization mode (ESI+) on the same C18 column (Waters UPLC BEH C18-2.1 x 100 mm, 1.7um) but with separate reversed-phase ultra-high-performance liquid chromatography-tandem mass spectrometry (RP/UPLC-MS/MS) methods: one optimized for hydrophilic compounds and one optimized for hydrophobic compounds. The third aliquot was analysed under basic conditions with a different RP/UPLC-MS/MS method operating in negative ESI mode (ESI-), using methanol, water, and 6.5 mM Ammonium Bicarbonate at pH 8.0 to elute biochemicals from a separate dedicated C18 column (Waters UPLC BEH C18-2.1 x 100 mm, 1.7um). The fourth aliquot was analysed on a hydrophilic interaction chromatography (HILIC)/UPLC-MS/MS method in ESI- after gradient elution from a HILIC column (Waters UPLC BEH Amide 2.1 x 150 mm, 1.7 um) with water and acetonitrile with 10mM Ammonium Formate, pH 10.8. All four methods utilized Waters ACQUITY ultra-performance liquid chromatography (UPLC) systems and Thermo Scientific Q-Exactive high resolution/accurate mass spectrometers interfaced with heated electrospray ionization (HESI-II) source and operated at 35,000 mass resolution.

### Quality control and compound identification

Compounds were identified by comparing the mass-to-charge (m/z), retention time, and associated fragmentation spectra in each sample to an in-house library of authentic standard chemical entities as described [13–15]. Compounds marked with an “*” after the name are MSI level 2 confidence identifications, all others are MSI level 1 confidence identifications [16]. One metabolite, 1-oleoyl-GPC (O-18:1), was removed from Run 2 data as it had been added to Metabolon’s library after Run 1 (though detection in Run 1 was retrospectively confirmed in all samples).

### Statistical analysis

First, to evaluate the reproducibility of the data after 3 years of storage, each data set was independently batch-normalized (for each batch within each data set, for a given metabolite, divide its raw peak areas by the median of the raw peaks areas of that metabolite in that batch) and imputed (after normalization, the missing values for a metabolite were imputed with the observed minimum). Principal Components Analysis (PCA) was performed on each set separately. Additionally, pairwise Welch two-sample t-tests comparing breeds were performed on the (natural) log-transformed data for each data set separately. Classification Analyses on dog breeds were performed separately for each data set with (1) Random Forest analysis [17] and (2) Partial Least Squares Discriminant Analysis (PLS-DA).

Next, the two data sets were merged with two different methods: (1) stacking the batch-normalized, imputed data sets (excluding metabolites not present in both) and (2) normalizing each batch within each data to the DBS QC samples (for a metabolite, divide each raw peak areas by the median raw peak area of its QC samples in that batch. Note the metabolite had to be present in at least two-thirds of the QC samples in that batch to be included), stacking the resulting data sets, and then imputing across the merged set. Metabolites that were not present in both sets or could not be QC-normalized in every batch were dropped. Hierarchical Clustering Analysis (HCA) was performed for both merged data sets excluding the cat samples to determine how well the same dogs clustered. HCA was performed in R using complete linkage with Euclidean distance on the log-transformed data.

For a more robust test of the ability to merge different data sets using bridging samples, a random number generator was used to create a synthetic data set from the merged QC-normalized data with each group of dog breeds having five different animals selected from each of the two individual data sets (“Merged Set 1”). The complementary merged set was also created (“Merged Set 2”). The goal was to mimic what would happen if half the samples were sent for Set 1 and the remaining samples were sent for Set 2. Random Forest Analysis was performed for both synthetic data sets.

The Welch two-sample t-tests were performed using Metabolon’s internal statistics pipeline, which uses R [18]. To account for multiple comparisons, q-values were computed using the method of Storey and Tibshirani, which provides estimates of the False Discovery Rates (FDRs) for each possible p-value cutoff [19]. The q-values were computed with Metabolon’s internal statistics pipeline using the “qvalue” R package. The random forest was run with the “randomForest” package [20]. The parameters for the random forest: sampled equally from each group without replacement, used the default *mtry* parameter (square root of the number of metabolites), and 50,000 trees. Hierarchical clustering was also performed with R on the log-transformed data with complete clustering using Euclidean distances.

Principal Components Analysis (PCA) and Partial Least-Squares Discriminant Analysis (PLS-DA) were performed in Metabolon’s Bioinformatics portal [21]. PCA was performed on each independent data set with all samples included and with only dogs, using the log-transformed data, and on the merged QC-normalized data set with all samples included. PLS-DA was performed on independent data sets with only the dog groups included. A cross-validation was performed for each independent PLS-DA using the leave-one-out CV (LOOCV) method and 4 PLS-DA components.

## Results and discussion

Data quality was assessed by determining the variability of both the internal standards and endogenous metabolites in the DBS QC samples. The internal standards (IS) demonstrated 2% and 4% median relative standard deviation in Run 1 and Run 2, respectively, related to instrument variability (IS were spiked after extraction but prior to instrument analysis), and the total process variability (includes extraction variability), based on endogenous biochemicals from technical replicates of pooled QC samples had a median RSD of 9% both runs.

### Independent analyses show similar biology and metabolomic profiles after 3 years of storage

First, to assess agreement between the two data sets, the correlations of the metabolites between the two sets were computed. The median correlation of the metabolites that were present in at least 50% of the samples in both data sets was 0.94 (n = 611). Because the cats might have be driving much of this correlation, the computations were also performed using just the samples from the dogs. For metabolites that were present in at least 50% of the dog samples in both sets, the median correlation was 0.90 (n = 536).

**Fig 2:**
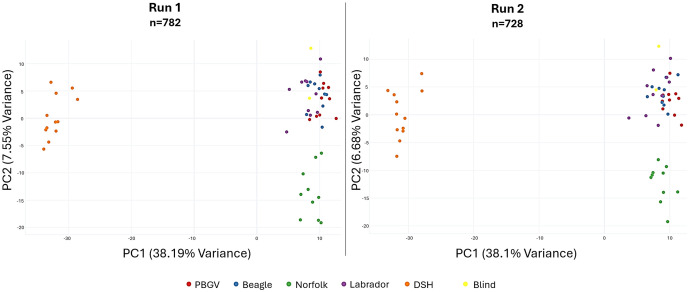
PCA of all samples. Data shows similar separation of cat (DSH) from dogs in both runs along Principal Component 1 and between small (Norfolk) and other dog breed sizes along Principal Component 2.

The number of metabolites reported in the two data sets was 782 (Run 1) and 728 (Run 2) (S1 and S2 Tables). Despite this 7% loss, an initial overview of the data using multivariate analysis showed that the variances in the first two principal components were visually very similar in the two runs (Fig 2). The first two PCs also provided similar interpretation, with clear separation between cats (DSH) and dog breeds in PC1, and between the small breed (Norfolk terrier) and other breeds in PC2 (Fig 2) in both data sets. The correlations of the principal components scores were also very high: the correlations between the first, second, and third principal components scores were 0.999, 0.927 and 0.955, respectively.

**Fig 3:**
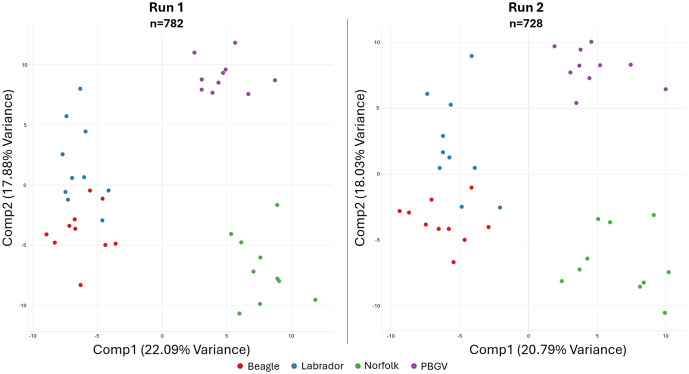
PLS-DA of all dog samples. Data shows similar separation of Norfolk terriers and Petit Basset Griffon Vendeens (PBGV) from Beagles and Labrador retrievers along Principal Component 1, and of Norfolk terriers and Beagles from Labradors and PBGVs along Principal Component 2.

PLS-DA is often used to identify biomarkers discriminating between classes. Using dog breed as class, the two independent runs visually gave similar results (Fig 3), with similar separation between breeds. Cross-validation of each independent PLS-DA model showed similar predictive ability, with 97.5% accuracy in both.

We also applied a random forest (RF) analysis to each data set separately to classify dog samples by breed, then compared the classifications across runs. For both sets, all dogs except one Beagle were classified correctly and predictive probabilities were similar between the two sets (the Out-of-Bag (OOB) Error = 2.5% for each set). One Beagle was misclassified as PBGV in set 1 (probability 0.32 vs 0.35), and a different Beagle was misclassified as Labrador in set 2 (probability 0.30 vs 0.31). Notably, the two blinded samples were correctly classified as Beagles in both sets with similar success (Table 1). The similarity in predictive probabilities and correct classifications in both sets indicates that storage for 3 years at −80°C had no significant impact on the conclusions drawn from a random forest analysis in this data set.

**Table 1:** Predictive probabilities from a random forest analysis applied to each data set separately correctly predicts blinded samples as Beagles in both sets.

Run 1							
Sample ID	Beagle		Labrador		Norfolk		PBGV
Blind 1	0.43		0.20		0.09		0.28
Blind 2	0.38		0.27		0.07		0.27
Run 2							
Sample ID	Beagle		Labrador		Norfolk		PBGV
Blind 1	0.43		0.23		0.10		0.24
Blind 2	0.37		0.31		0.08		0.24

**Fig 4:**
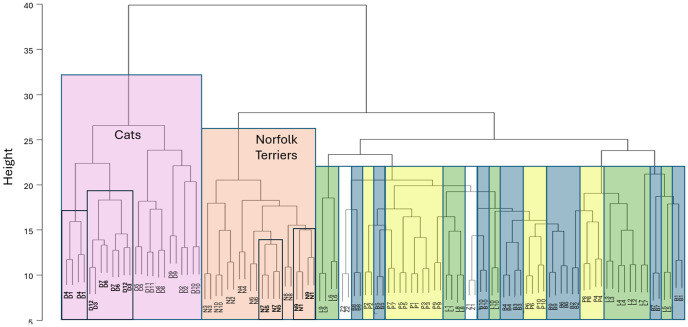
HCA of stacked batch-normalized and independently imputed data sets. Domestic Shorthair cat (D, pink) and Norfolk terrier (N, orange) formed distinct clusters. Labrador retriever (L, green), Petit Basset Griffon Vendeen (P, yellow) and Beagle (B, blue) did not. Both blinded sample’s (Z, white) nearest neighbours were Beagles. The number ID depicts an individual. Black boxes indicate cases where individuals did not pair.

To test for consistency/reproducibility of the data in two different runs we performed a Hierarchical Clustering Analysis (HCA) on the stacked batch-normalized, imputed data (Fig 4). Of the 54 possible pairs, 44 (81%) paired correctly and the remaining 10 reciprocally paired within the same breed/species. While the medium and large dogs did not cluster by breed, like Norfolk terrier and DSH did, all subjects paired within the correct breed, illustrating that both runs shared a similar challenge in clearly differentiating breeds of similar size. The data show that after 3 years of storage the biological separations are highly conserved to the level of individuals within each breed clustering together consistently. This may be used to suggest that any alterations to biochemical signatures due to storage are minimal in comparison to biological diversity in the population within this data set.

### Composition of detected metabolites in separate analyses

Despite biological separations being highly conserved, we wanted to understand the cause of differences in the detected metabolome and to determine whether any specific metabolite class was prone to instability. In the study 55 metabolites were novel to Run 1, while none were novel to Run 2. A reduction in the numbers of metabolites detected between the analysis of the same samples three years apart can have several potential sources, including loss of metabolites due to degradation and instrument sensitivity differences. Notably, while punch location (e.g., middle of the spot or off-center) can introduce variability when spot size varies widely within a study, this source was ruled out based on all spots containing exactly 20uL of blood and Metabolon’s standard operating procedure that all punches were taken from the middle of each spot (to mitigate sources of variation). To assess the relative contribution of the two more likely factors, data were first reviewed for compounds historically observed to be susceptible to degradation, then for detection rate and overall signal intensity differences between the analyses. Compound degradation tends to be tied to compound class/pathway rather than equally distributed across class, while in the case of instrument sensitivity differences, compounds present at lower levels (lower detection percentage and/or lower signal intensities) tend to be more heavily impacted and spread across compound class rather than restricted to specific classes. Detection differences related to variable extraction efficiencies between runs were ruled out based on the same DBS extraction method SOP followed for each analysis and high precision of technical replicates demonstrating reproducible extraction methodology.

Comparing those metabolites only detected in Run 1, they were not restricted to specific classes but covered a range of metabolite classes (Table 2). The net difference varied from 0 to 13% per class, with a range of 0–27 metabolites (Table 2), contributing to a negligible overall shift in class distribution.

**Table 2:** The number of metabolites reported in each analysis with net reduction and proportion per Super Pathway.

	Number of Metabolites		Net Difference	
Summary	Run 1	Run 2	N	%
Total Metabolites	783	728	55	7.3
Total Named Metabolites	730	678	52	7.4
By Super Pathway				
Lipid	297	270	27	9.5
Amino Acid	199	183	16	8.4
Xenobiotics	65	63	2	3.1
Nucleotide	60	57	3	5.1
Carbohydrate	36	35	1	2.8
Peptide	29	28	1	3.5
Cofactors and Vitamins	28	28	0	0.0
Energy	8	7	1	13.3
Partially Characterized Molecules	8	7	1	13.3
Unnamed	53	50	3	5.8

To better understand the reason for the detection differences we made frequency plots of raw peak areas in Run 1 of those metabolites absent from Run 2. We identified that 80% (44) of the 55 metabolites were present on the lower end of the peak area distribution in Run 1 (S1 Fig). This indicates that factors such as instrument sensitivity differences at baseline, were probably a driving factor for the reduction of detected compounds. This interpretation is supported by the reduced signal observed for an independent matrix type, human plasma, QC sample in Run 2 as compared to the same human plasma QC sample in Run 1 (S2 Fig).

Univariate analysis to identify metabolites that differed in pairwise comparisons with statistical significance (p < 0.05) was also undertaken. Despite the reduction in the numbers of metabolites that met the criteria in Run 2 (range 1–66), the percent metabolome that met statistical significance was within 1–4% between the two runs (Table 3).

**Table 3:** Univariate analysis of pair-wise comparisons in the two runs.

		Pair-wise Comparisons									
		D P	D B	D N	D L	N P	N L	N B	L P	L B	B P
	Total # of metabolites	# of metabolites that met statistical significance (p < 0.05)									
Run 1	782	575	565	564	549	290	283	270	197	147	111
Run 2	728	531	499	515	503	254	247	233	176	144	110
Net loss (#) of significant metabolites in Run 2		44	66	49	46	36	36	37	21	3	1
Net loss (%) of significant metabolites in Run 2		8	12	9	8	12	13	14	11	2	1
# of significant metabolites in both runs		492	475	486	469	209	211	199	143	111	65
	Total # of metabolites	% of metabolites that met statistical significance (p < 0.05)									
Run 1	782	74	72	72	70	37	36	35	25	19	14
Run 2	728	73	69	71	69	35	34	32	24	20	15
Change in % metabolites meeting statistical significance		−1	−4	−1	−1	−2	−2	−3	−1	1	1

The number of metabolites, the percent metabolome and the net change between runs that met the criteria independently and identified in both runs are reported. Domestic Shorthair cat (D), Norfolk Terrier (N), Labrador retriever (L), Petit Basset Griffon Vendeen (P), and Beagle (B). Percent (%). Number (#).

Even with variable detection rates, the biological differences between groups overcame run-to-run differences. Based on this overview, it is possible to consider two DBS metabolome analyses separated by 3 years as providing comparable data suitable for interpretation.

This data builds upon previous reports of the DBS metabolome, which observed metabolome stability over different time periods (within or inter-batch) without comparing the utility of the data for class discrimination [8–10,22].

### Evaluating the ability to merge canine DBS sets using a human DBS QC

**Fig 5:**
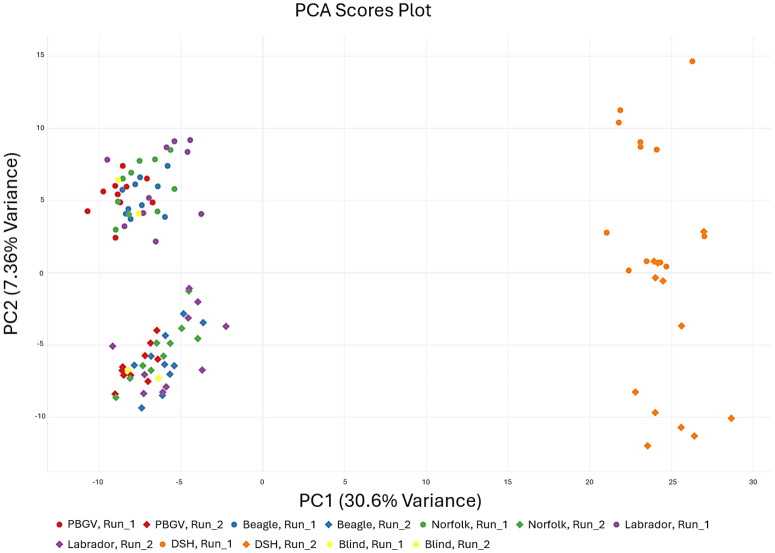
PCA of all samples in the QC-normalized merged data set with 561 metabolites. Run 1 (circles) and Run 2 (diamonds) show similar separation of cats (DSH) from dogs along Principal Component 1, but a batch-effect was observed along Principal Component 2.

**Fig 6:**
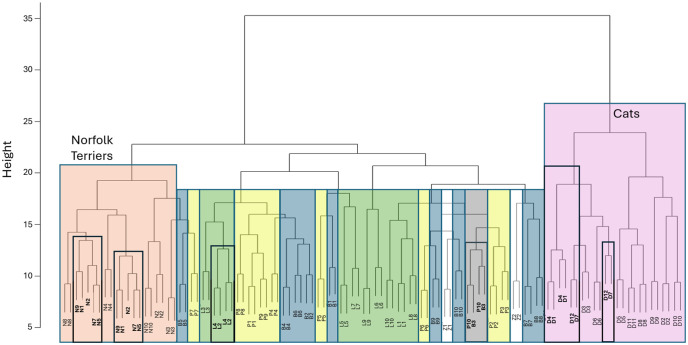
HCA on merged data normalized to a human DBS QC sample. Domestic Shorthair cat (D, pink) and Norfolk terrier (N, orange) form single clusters while the other breeds do not (Labrador retriever (L, green), PBVG (P, yellow), Beagle (B, blue and Z, white)). The number ID depicts an individual. Black boxes indicate cases where individuals did not pair within breed. Gray box indicates two different breeds paired at individual level.

A second objective was to develop a method to bridge the data to allow different experiments to be interoperable and gain additional outputs/insights (a very useful tool not only in biobank samples but in all experimental studies). We normalized metabolites common to both data using the human DBS QC samples, resulting in 561 metabolites in the merged data set. A PCA of the merged data demonstrated that the primary separation in variance along Principal Component 1 remains similar due to the cats being very different from dogs, but there was a shift along Principal Component 2 indicating a batch effect accounting for 7.4% of the variability (Fig 5). Even with this noted batch difference HCA still demonstrated good accuracy (Fig 6) with 41 of the 54 potential pairs being paired correctly (76%), 10 further reciprocally paired within the same breed/species, two reciprocally paired with different breeds (P10 and B3) of the same size, and one individual Norfolk (N6) that did not pair, though both replicates accurately clustered with the other Norfolk terriers.

To further test the suitability of merging different data sets using bridging samples to still correctly predict a blinded sample, we created two synthetic data sets from the merged set using a random number generator so that each breed in the synthetic data set has 5 animals from each of the two individual sets (the other synthetic data set is the complement of this). Random forest analysis was performed separately on each of the two synthetic data sets. The OOB Error for Set 1 was 10%, and for Set 2 it was 7.5%. These random forests were also used to predict the breed of the blinded samples (Table 4). The merged data sets proved able to predict the correct breed with good confidence in six of eight comparisons, with the other two comparisons being a tie between the correct breed or one other with similar probabilities.

**Table 4:** Random Forest predictions of breed for two blinded samples using two data sets derived from merged data and normalized through a bridging sample, shown alongside the predictions using each run separately from Table 1.

			RF predicted breed for blinded samples			
Animal ID	Run ID	Data set used for modelling RF	Beagle	Labrador	Norfolk	PBGV
Blind 1	Run 1	RUN 1	**0.43**	0.20	0.09	0.28
	Run 1	Merged Set 1	**0.45**	0.22	0.09	0.24
	Run 1	Merged Set 2	**0.40**	0.19	0.10	0.31
	Run 2	RUN 2	**0.43**	0.23	0.10	0.24
	Run 2	Merged Set 1	**0.37**	0.26	0.10	0.27
	Run 2	Merged Set 2	**0.40**	0.22	0.12	0.27
Blind 2	Run 1	RUN 1	**0.38**	0.27	0.07	0.27
	Run 1	Merged Set 1	**0.37**	0.30	0.07	0.26
	Run 1	Merged Set 2	**0.31**	0.28	0.08	**0.32**
	Run 2	RUN 2	**0.37**	0.31	0.08	0.24
	Run 2	Merged Set 1	**0.34**	**0.34**	0.07	0.25
	Run 2	Merged Set 2	**0.36**	0.29	0.07	0.29

## Conclusion

Metabolomics is a powerful analytical approach for biomarker discovery from DBS samples, offering unique biochemical insights by providing a comprehensive view of underlying biological processes present in both plasma and blood-borne cells. The objectives for this proof of principle work were to establish whether DBS samples are sufficiently stable such that, after 3 years of storage at −80°C, the ability to successfully discriminate between different phenotypic classes using metabolomics data could be maintained. This objective and associated study design are most aligned with biobanking strategies, which aim to analyse and store samples for analysis overtime. Two separate approaches were investigated to analyse the data. First, whether analyzing the two data sets (Run 1 and Run 2) independently provided similar biological stratification information after 3 years of storage and secondly, to determine whether it was possible to combine the metabolomics data from separate runs analysed 3 years apart to establish a precedent for data analysis over longitudinal data sets, for example to facilitate insights from prospective biobanks.

Despite the overall DBS metabolome being ~7% smaller in the second run, comparisons between the two runs demonstrated similar abilities to observe variance associated with phenotypes (species and breed size) and to correctly identify the breed of blinded samples using PLS-DA and RF with similar predictive probabilities. Many highly ranked metabolites used to aid classification by both methods were also consistent between the two data sets. Furthermore, cluster analysis showed very good alignment of the samples between runs demonstrating that there are greater similarities than differences between the two data sets. Univariate analysis of pairwise comparisons identified fewer metabolites met the statistical significance threshold (p < 0.05) in Run 2, but they remained proportionate to the size of the metabolome and enabled similar interpretation.

A series of analyses demonstrated that the loss of metabolites from the metabolome following three years of storage was unlikely to be due to chemical instability/degradation in most cases. For example, no increases in detected oxidized lipids, such as long-chain mono- and poly-unsaturated fatty acids, were identified, nor any novel metabolites in Run 2 that might represent degraded products. Instead, evidence indicates that the major factor contributing factor was instrument variability in sensitivity thresholds, which can occur irrespective of storage time and is unlikely to impact biochemical interpretation. Consistent with this interpretation, most compounds missing from Run 2 were present at lower intensities with potential to be on the boarder of limit of detection depending on run-day. Further, the compounds in the second analysis span a wide range of compound classes rather than being focused on specific degradation-sensitive classes. We use these observations to propose that DBS samples have minimal loss of information due to storage for up to 3 years and are sufficiently stable to be used for metabolomic studies.

An additional source of variability between DBS samples is the selection of the punch sample when blood is not evenly distributed on the card [23]. In this study we sampled duplicate spots from small blood aliquots (20 µL). We would recommend aliquoting blood to reduce variability between DBS samples in a biobank and obtaining, where possible, additional data for normalization, such as hematocrit. Larger volume aliquots were not tested as part of this study, which may improve numbers of metabolites that are detected but may also increase variability in the blood surface area/drying time [24].

A consideration for comparisons made between these two independent runs is that similar factors affect all samples within a batch. The study design did not enable us to compare a cohort of DBS samples stored across 3 years within the same instrument analysis batch as might occur in a retrospective biobank study. However, we were able to consider how a prospective biobank analysis might operate, by assessing the success of a merging strategy of the data from the two independent analyses. One challenge to comparing independent metabolomic studies is that most are semi-quantitative, where samples are quantified relative to the pool for that population. The two data sets were “merged” by normalizing both data sets using an independent human DBS quality control sample present in both analyses. We demonstrate successful interoperability between data sets extracted years apart, with a minimal reduction from 81 to 76% alignment of identical samples in HCA between the independent and merged data sets (Fig 4 vs Fig 6). In addition, the development of two data sets, each spanning samples extracted 3 years apart, identified blinded samples with good confidence, despite the normalization of a metabolome limited to those metabolites detected in human DBS too. In future studies, the inclusion of a larger “blinded sample” cohort covering other classes within the study design would assess the success of the merger with greater confidence. The merged data showed that bridging with a human DBS QC is a feasible option to re-utilize previously acquired data sets. However, for researchers planning re-use of metabolome data (such as in prospective, longitudinal studies), we recommend inclusion of the most relevant bridging sample a priori to the first analysis. For example, in the study presented here, a pooled feline/canine DBS QC would likely have aligned the data sets better. The approach also has applications beyond DBS studies as it demonstrates that quality control matrices can act as a normalization method to combine multiple independent metabolomics studies over time and can be deployed retrospectively, with improved biomarker discovery. This may accelerate research and enable reutilization of metabolome data to other areas of research, extracting additional biological and economic value from metabolomics studies.

### Strengths and limitations

The primary objective of this study was to understand DBS stability within the operational underpinning of research relying on biobanked samples collected from remnant blood in veterinary clinics. The study design was established such that the exact same samples could be analysed three years apart. This design removed confounding biological factors related to separate sampling, either a second draw from the same animals or an independent cohort of animals. By analyzing the same exact samples, the only variability in the analysis would therefore arise from storage and analytical batch analysis factors. In our experience biological variability is much larger than analytical variability, so repeat sampling of the same animals or gathering a new cohort of samples would add larger confounding variables than what we expected to encounter from analytical factors. This permitted a more precise assessment of the impact of the storage parameter.

This choice did introduce the complexity of having to account for the variability associated with instrument performance differences overtime. To address this, we analysed the samples using two strategies. The first was to analyse the two analyses (T0 and T3 year) independently to see if biological stratification was conserved. The years of storage demonstrated minimal impact on the ability to draw the same conclusions from the samples. While this analysis removed much of influence of instrumentation differences, we also wanted to assess if the data could be stitched together into one dataset utilizing an external normalization sample. This analysis showed that the normalization strategy maintained most of the biological stratification of the data but was not fully corrected for the batch effects. We hypothesize that some of this was due to the use of a non-species matched DBS sample for normalization. The choice of a human DBS bridging sample was based on practicality. Procuring a significant volume of pooled human whole blood was possible, where it would have been much more difficult to obtain a large pool of whole blood from dogs/cats. However, it should be stated that if this analysis becomes needed and routine within animal biobanks, efforts should be taken to obtain such quality control samples.

## Supporting information

S1 Fig Histogram of median raw peak area in Run 1 for 55 metabolites not detected in Run 2. This supports the interpretation that the lack of detection in Run 2 was likely to be due to data being below the level of detection in most cases, rather than degradation with time.
(DOCX)

S2 Fig Example of the reduction in signal intensity for a metabolite reduced in Run 2 compared to Run 1, including that of a human plasma QC sample. This supports the proposal that instrument sensitivity rather than instability in DBS caused the lower detection rate observed in Run 2.
(DOCX)

S1 Table Data Tables from Run 1. (XLSX)

S2 Table Data Tables from Run 2. (XLSX)
