# Supplementary material for: Dried blood spot 3-year stability suitability for biomarker discovery and biobanking initiatives in companion animals
Source: PLoS One. 2026 Aug 11;21(8):e0352031. doi: 10.1371/journal.pone.0352031 (PMC13460606; doi:10.1371/journal.pone.0352031)
Supplement: Supplementary file 1 [file pone.0352031.s001.docx]

**S1 Fig. Histogram of median raw peak area in Run 1 for 55 metabolites not detected in Run 2.**  This supports the interpretation that the lack of detection in Run 2 was likely to be due to data being below the level of detection in most cases, rather than degradation with time.
