# Supplementary material for: Dried blood spot 3-year stability suitability for biomarker discovery and biobanking initiatives in companion animals
Source: PLoS One. 2026 Aug 11;21(8):e0352031. doi: 10.1371/journal.pone.0352031 (PMC13460606; doi:10.1371/journal.pone.0352031)
Supplement: Supplementary file 2 [file pone.0352031.s002.docx]

**S2 Fig. Example of the reduction in signal intensity for a metabolite reduced in Run 2 compared to Run 1, including that of a human plasma QC sample.** This supports the proposal that instrument sensitivity rather than instability in DBS caused the lower detection rate observed in Run 2.
